# Association of MMP-2, RB and PAI-1 with decreased recurrence-free survival and overall survival in bladder cancer patients

**DOI:** 10.18632/oncotarget.20686

**Published:** 2017-09-06

**Authors:** Owen T.M. Chan, Hideki Furuya, Ian Pagano, Yoshiko Shimizu, Kanani Hokutan, Lars Dyrskjøt, Jørgen Bjerggaard Jensen, Per-Uno Malmstrom, Ulrika Segersten, Filip Janku, Charles J. Rosser

**Affiliations:** ^1^ Clinical and Translational Research Program University of Hawaii Cancer Center, Honolulu, HI, USA; ^2^ Cancer Prevention and Control Program Research Program University of Hawaii Cancer Center, Honolulu, HI, USA; ^3^ Department of Molecular Biosciences and Bioengineering, University of Hawaii at Manoa, Honolulu, HI, USA; ^4^ Department of Molecular Medicine, Aarhus University Hospital, Aarhus, Denmark; ^5^ Department of Urology, Aarhus University Hospital, Aarhus, Denmark; ^6^ Departments of Surgical Sciences, Uppsala University, Uppsala, Sweden; ^7^ Department of Investigational Cancer Therapeutics, University of Texas MD Anderson Cancer Center, Houston, TX, USA

**Keywords:** angiogenin, bladder cancer, IHC, MMP-2, PAI-1

## Abstract

**Background:**

We previously reported an accurate urine-based bladder cancer (BCa)-associated diagnostic signature that can be used to non-invasively detect BCa. In this study, we investigated whether a component of this signature could risk stratify patients with BCa.

**Methods:**

Utilizing immunohistochemistry, we investigated angiogenin, MMP-2, p53, RB and PAI-1 expression from 939 patients with BCa. The expression levels were scored by assigning a proportion score and an intensity score to yield a total staining score for each protein. The expressions of each protein individually and as an aggregate were then correlated with progression-free survival (PFS), cancer-specific survival (CSS) and overall survival (OS).

**Results:**

Differential expressions of these markers were noted in BCa. With multivariate analysis in non-muscle invasive bladder cancer (NMIBC) age, tumor grade portended a worse PFS, while age, tumor grade, nodal status, MMP2, RB and PAI-1 expression portended a worse OS. As for multivariate analysis in muscle invasive bladder cancer (MIBC), age MMP-2 and RB were associated with a worse PFS, while age, nodal status, MMP-2, RB and PAI-1 were associated with a worse OS. Using Kaplan-Meier survival analysis, we noted a significant reduction in OS as more of the five biomarkers were expressed in a tumor. Thus, overall, high expressions of MMP-2, RB and/or PAI-1 in bladder tumors were markers of poor prognosis.

**Conclusion:**

Individually, MMP-2, RB and PAI-1, as well as in aggregate correlated with poor survival in patients with BCa. Thus, patients whose bladder tumors express these biomarkers may benefit from early radical treatment and/or neoadjuvant or adjuvant therapies.

## INTRODUCTION

Cancer of the urinary bladder is the fourth most common malignancy in men and the ninth most common malignancy in women in the United States [1]. Urothelial carcinomas constitute approximately 90% of all bladder cancer (BCa) cases [2]. At presentation, more than 80% of bladder tumors are non-muscle invasive bladder cancer (NMIBC, *i.e.,* Tis, Ta or T1) and the remaining 20% of bladder tumors are muscle-invasive bladder cancers (MIBC) or metastatic. NMIBC harbors a 5-year survival rate of approximately 94% [3, 4]; however, approximately 70% of patients with these lesions develop tumor recurrence within two years of initial diagnosis. The recurrence phenomenon of NMIBC makes it one of the most prevalent cancers worldwide; in America, it is second only to colorectal cancer. Therefore, NMIBC is a significant burden to healthcare systems [1, 5]. As for MIBC, 5-year survival rate is approximately 50%, which drops off to < 20% in the case of metastatic disease [6, 7].

Methods to identify NMIBC and MIBC patients, who are at increased risk of disease recurrence and succumbing to disease, have previously relied on conventional histopathologic evaluation. These histopathologic features have failed to properly risk stratify these patients. Thus, prognostic markers are urgently needed to identify high-risk patients who would benefit from early radical treatment and/or neoadjuvant or adjuvant therapies.

In our previous studies, we have coupled high-throughput, discovery-based technology (*i.e.,* genomics and proteomics) with bioinformatics, in order to derive a BCa-associated diagnostic signature that shows promise for the accurate detection of BCa in voided urine samples [8–11]. In five independent studies, we have validated our 10 biomarkers, which comprise our BCa-associated diagnostic signature [12–16]. Additionally, we have reported the prognostic significance of two of the 10 biomarkers that comprise the BCa-associated diagnostic signature, angiogenin (ANG) and plasminogen activator inhibitor-1 (PAI-1) [17–20]. Furthermore, in-depth molecular analyses of ANG and PAI-1 have identified other key molecules that possess a significant downstream or upstream interplay with them (*i.e.,* MMP-2 and p53 with ANG and RB and p53 with PAI-1) [17-19, 21].

In the present study, we set out to validate the tissue expression of ANG, MMP-2, p53, PAI-1 and RB in human BCa with immunohistochemistry conducted on tissue microarrays (TMA). Demographic, clinical, disease and treatment characteristics are presented in Table 1. The aims of the validation were to a) analyze the tissue immunoreactivity of these BCa candidate biomarkers and b) investigate if the expression of these BCa candidate biomarkers possesses prognostic value in determining recurrence-free survival and overall survival in BCa patients.

**Table 1:** Demographic, clinical, and pathologic characteristics of the 939 subjects comprising the study cohort

Features	Denmark N = 587	Sweden N = 352	All Patients N = 939
Age (years)			
≤ 65	274 (47%)	90 (26%)	364 (39%)
> 65	313 (53%)	256 (73%)	569 (61%)
Unavailable	0 ( 0%)	6 ( 2%)	6 ( 1%)
Gender			
Female	144 (25%)	85 (24%)	229 (24%)
Male	443 (75%)	264 (75%)	707 (75%)
Unavailable	0 ( 0%)	3 ( 1%)	3 ( 0%)
Tumor Grade			
Low	139 (24%)	84 (24%)	223 (24%)
High	437 (74%)	265 (75%)	702 (75%)
Unavailable	11 ( 2%)	3 ( 1%)	14 ( 1%)
Tumor Stage			
Ta or Tis	139 (24%)	119 (34%)	258 (27%)
T1	133 (23%)	117 (33%)	250 (27%)
T2	294 (50%)	89 (25%)	383 (41%)
T3 or T4	21 ( 4%)	23 ( 7%)	44 ( 5%)
Unavailable	0 ( 0%)	4 ( 1%)	4 ( 0%)
Lymph Nodes			
N0 or Nx	470 (80%)	341 (97%)	811 (86%)
N1	117 (20%)	11 ( 3%)	128 (14%)
Progression			
No	318 (54%)	230 (65%)	548 (58%)
Yes	269 (46%)	110 (31%)	379 (40%)
Unavailable	0 ( 0%)	12 ( 3%)	12 ( 1%)
Follow-up (years)			
Median	6.83	3.75	5.37

## RESULTS

### P53, RB and PAI-1 are preferentially associated with aggressive bladder cancer

**Figure 1:**
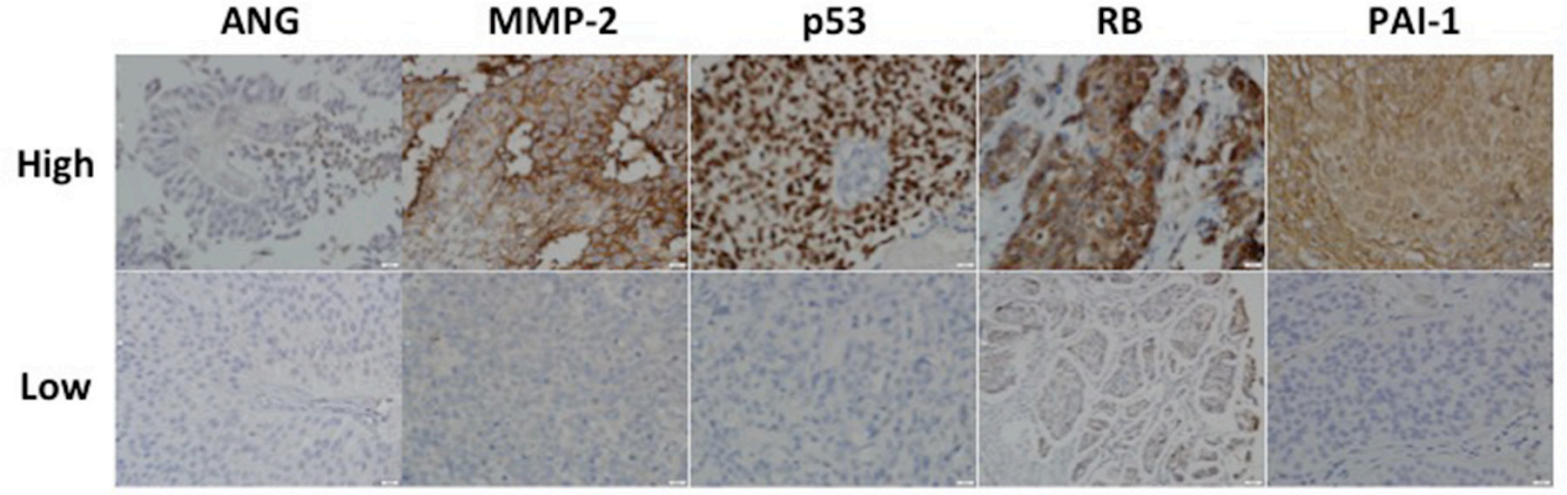
Representative expression status for ANG, MMP-2, p53, RB and PAI-1 in a high-grade non-muscle invasive bladder cancer Upper row represents high intensity while lower row represents low intensity associated with each target. All images were captured at 400× magnification.

Figure 1 shows representative immunohistochemical stainings for each of the five targets in a high-grade non-muscle invasive tumor. The relationship between immunophenotype for each target and tumor grade is summarized in Table 2. We found the highest expression levels of p53, RB and PAI-1 (total staining score ≥3) associated with high-grade disease compared to low-grade disease (58% *vs.* 27%, 85% *vs.* 65%, 65% *vs.* 49%, respectively). Furthermore, the highest expression of MMP-2 was noted with low-grade disease compared to high-grade disease (64% *vs.* 52%). These differences were statistically significant. The relationships between immunophenotype for each target and tumor stage are summarized in Table 3. We found the highest expression levels of p53 and RB in T2 stage tumors (compared to Ta/Tis stage tumors (62% *vs.* 29% and 87% *vs.* 68%, respectively). Similar to its expression in low-grade tumors, MMP-2 expression was higher in Ta/Tis stage tumors compared to T2 stage tumors (62% *vs.* 47%). These differences were statistically significant. However, we observed no difference in p53, RB and MMP-2 expression between T2 and T3/T4 stage tumors.

**Table 2:** Relationship between immunochemical features and tumor grade

Target Expression	Low-Grade N (Column %)	High-Grade N (Column %)	*p*-value^*^
ANG			
0-2	181 (93%)	547 (92%)	.64
3-4	13 ( 7%)	48 ( 8%)	
5-6	0 ( 0%)	0 ( 0%)	
MMP-2			
0-2	75 (36%)	286 (48%)	.01
3-4	129 (63%)	306 (51%)	
5-6	2 ( 1%)	6 ( 1%)	
p53			
0-2	158 (73%)	270 (42%)	<.0001
3-4	55 (25%)	301 (46%)	
5-6	5 ( 2%)	78 (12%)	
Rb			
0-2	46 (23%)	89 (15%)	.008
3-4	152 (76%)	508 (83%)	
5-6	1 ( 1%)	13 ( 2%)	
PAI-1			
0-2	108 (51%)	228 (35%)	<.0001
3-4	103 (48%)	400 (62%)	
5-6	2 ( 1%)	22 ( 3%)	

^*^Fisher’s Exact Test.

**Table 3:** Relationship between immunochemical features and tumor stage

Target Expression	Ta or Tis N (Column %)	T1 N (Column %)	T2 N (Column %)	T3 or T4 N (Column %)	*p*-value^*^
ANG					
0-2	212 (93%)	204 (94%)	283 (90%)	35 (92%)	.28
3-4	17 (7%)	12 (6%)	32 (10%)	3 ( 8%)	
5-6	0 (0%)	0 (0%)	0 (0%)	0 ( 0%)	
MMP-2					
0-2	91 (38%)	86 (38%)	170 (55%)	21 (55%)	<.0001
3-4	147 (61%)	140 (62%)	132 (43%)	17 (45%)	
5-6	2 (1%)	0 (0%)	6 (2%)	0 ( 0%)	
p53					
0-2	178 (71%)	112 (47%)	129 (38%)	11 (26%)	<.0001
3-4	68 (27%)	107 (45%)	160 (47%)	25 (58%)	
5-6	6 (2%)	18 ( 8%)	53 (15%)	7 (16%)	
Rb					
0-2	57 (24%)	40 (18%)	36 (11%)	3 ( 8%)	.0001
3-4	174 (75%)	181 (82%)	276 (86%)	35 (88%)	
5-6	2 (1%)	1 (0%)	9 (3%)	2 ( 5%)	
PAI-1					
0-2	109 (44%)	85 (36%)	133 (38%)	9 (23%)	.04
3-4	136 (55%)	147 (62%)	199 (57%)	30 (75%)	
5-6	4 ( 2%)	4 (2%)	15 (4%)	1 ( 3%)	

^*^Fisher’s Exact Test.

### MMP-2, p53, RB, PAI-1 are associated with a reduction in recurrence-free survival and overall survival in bladder cancer

**Figure 2:**
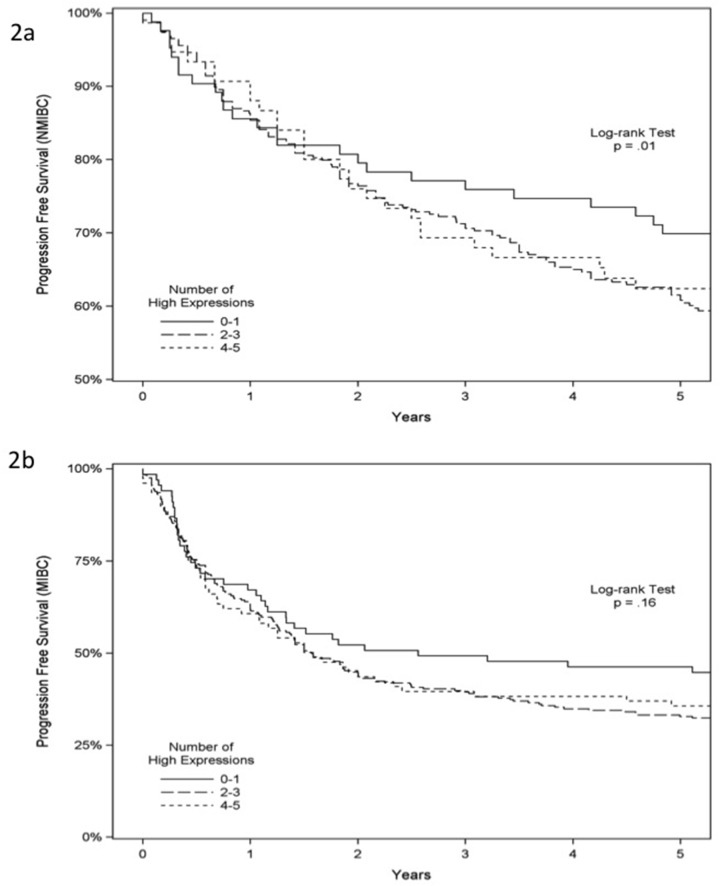
Progression-free survival analysis of 939 patients with bladder cancer Progression-free survival according to immunostaining status of ANG, MMP-2, p53, RB and PAI-1 in NMIBC **(A)** and MIBC **(B)**.

**Figure 3:**
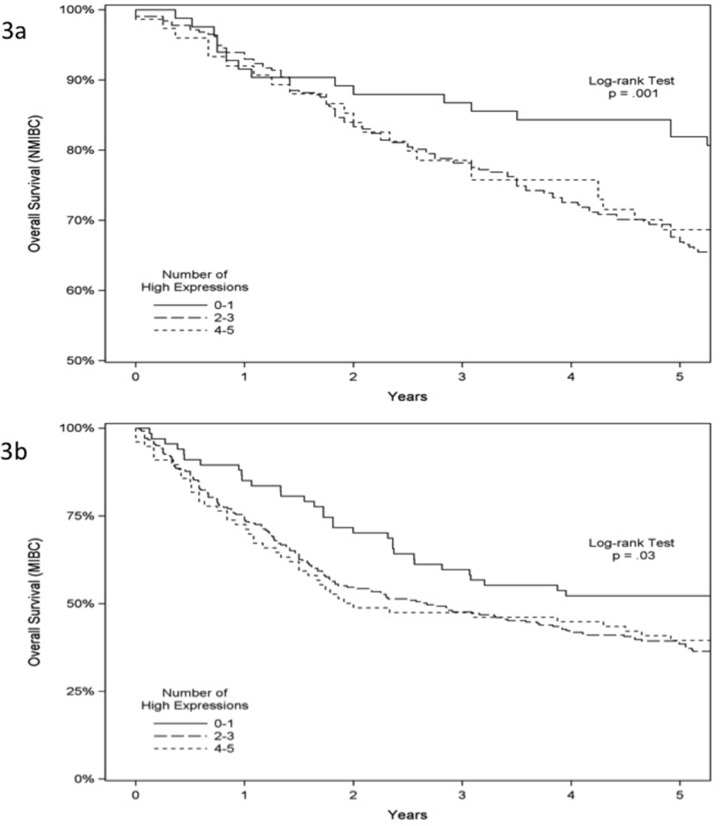
Overall survival analysis of 939 patients with bladder cancer Overall survival according to immunostaining status of ANG, MMP-2, p53, RB and PAI-1 in NMIBC **(A)** and MIBC **(B)**.

In NMIBC, univariate analysis indicated that age, tumor grade, tumor stage (Ta/Tis *vs.* T1), expression of p53 and RB predicted progression-free survival. With multivariate analysis, >65 years of age (HR 1.67, 95% CI 1.16-2.40, *p* = 0.006) and high-grade tumor (HR 1.74, 95% CI 1.04-2.92, *p* = 0.03) independently predicted a worse progression-free survival (Table 4a). In MIBC, univariate analysis found that age, gender, expression of MMP-2, RB and PAI-1 predicted progression-free survival. With multivariate analysis, >65 years of age (HR 1.26, 95% CI 1.00-1.58, *p* = 0.05), high MMP-2 expression (HR 2.03, 95%CI 1.28-3.22, *p* = 0.003) and high RB expression (HR 2.31, 95%CI 1.26-4.23, *p* = 0.007) independently predicted a worse progression-free survival (Table 4b). Using the Kaplan-Meier survival analysis with the log-rank test, we found significantly reduced progression-free survival in NMIBC but not in MIBC when more of the five biomarkers were expressed in a tumor (*p* = 0.01 and *p* = 0.16, respectively), Figure 2a and 2b. As noted in Table 4, three of the five biomarkers (MMP-2, RB and PAI-1) were critical biomarkers influencing survival analysis of NMIBC and MIBC. We noted no difference in cancer-specific survival in NMIBC or MIBC associated with the expression of these five biomarkers (Supplementary Figure 1a and 1b and Supplementary Table 1a and 1b). Lastly, in NMIBC, univariate analysis demonstrated that age, gender, tumor grade, tumor stage (Ta/Tis *vs.* T1) and expression of MMP-2, RB and PAI-1 predicted overall survival (OS). With multivariate analysis, >65 years of age (HR 2.41, 95% CI 1.78-3.26, *p* < 0.0001), high-grade tumor (HR 2.11, 95% CI 1.43-3.12, *p* = 0.0002) and high expression of MMP-2 (HR 1.87, 95% CI 1.08-3.26, *p* = 0.03), RB (HR 3.08, 95% CI 1.79-5.32, *p* = 0.0001) and PAI-1 (HR 2.58, 95% CI 1.52-4.38, *p* = 0.0004) independently predicted a worse OS (Table 5a). Similarly, in MIBC, univariate analysis revealed that age, gender, lymph nodes stage (N0/NX *vs.* N1) and expression of MMP-2, RB and PAI-1 predicted overall survival (OS). With multivariate analysis, >65 years of age (HR 1.38, 95% CI 1.10-1.74, *p* = 0.006), lymph nodes N1 stage (HR 1.40, 95% CI 1.0.9-1.80, *p* = 0.009) and high expression of MMP-2 (HR 2.08, 95% CI 1.29-3.33, *p* = 0.03), RB (HR 2.18, 95% CI 1.15-4.13, *p* = 0.02) and PAI-1 (HR 1.69, 95% CI 1.09-2.62, *p* = 0.02) independently predicted a worse OS (Table 5b), while male gender (HR 0.68, 95% CI 0.54-0.86, *p* = 0.002) independently predicted better OS. Using the Kaplan-Meier survival analysis with the log-rank test, we noted a significant reduction in OS in both NMIBC and MIBC as more of the five biomarkers were expressed in a tumor (*p* = 0.001 and *p* = 0.03, respectively), Figure 3a and 3b. Thus, the overall, high expression of MMP-2, RB and/or PAI-1 in bladder tumors was a marker of poor prognosis.

**Table 4a:** Progression Free Results for Non-Muscle Invasive Bladder Cancer

	Univariate Analyses^1^						Multivariate Analyses^1^					
	n	Median^2^	HR^3^	LCL	UCL	p^4^	n	Median^2^	HR^3^	LCL	UCL	p^4^
Age (years)												
≤ 65	183	41.7	1.00				181	36.8	1.00			
> 65	317	14.6	1.84	1.29	2.63	.0008	317	15.9	1.67	1.16	2.40	.006
Gender												
Female	95	21.2	1.00				95	18.2	1.00			
Male	405	22.1	0.98	0.66	1.46	.92	403	22.6	0.88	0.59	1.32	.54
Tumor Grade												
Low	219	50.5	1.00				219	36.2	1.00			
High	279	11.4	2.47	1.74	3.52	<.0001	279	14.5	1.74	1.04	2.92	.03
Tumor Stage												
Ta or Tis	253	44.6	1.00				252	27.8	1.00			
T1	247	11.0	2.34	1.68	3.27	<.0001	246	16.7	1.42	0.87	2.33	.16
Lymph Nodes												
N0 or N_X_	487	23.5	1.00				486	22.8	1.00			
N1	13	1.3	5.98	3.22	11.11	<.0001	12	2.8	4.25	2.20	8.21	<.0001
ANG												
0-2	408	25.6	1.00				408	24.3	1.00			
3-4	29	22.9	1.08	0.55	2.13	.83	28	26.2	0.97	0.47	2.00	.92
MMP-2												
0-2	175	26.1	1.00				173	25.5	1.00			
3-4	281	21.6	1.06	0.75	1.49	.75	281	20.4	1.09	0.77	1.55	.63
5-6	2	17.8	1.12	0.56	2.22		2	16.2	1.19	0.59	2.39	
p53												
0-2	284	29.8	1.00				284	23.6	1.00			
3-4	173	18.3	1.33	1.01	1.75	.04	173	21.5	1.08	0.81	1.44	.60
5-6	24	11.3	1.77	1.03	3.06		24	19.5	1.17	0.65	2.08	
Rb												
0-2	97	23.6	1.00				96	22.4	1.00			
3-4	347	27.4	0.93	0.62	1.40	.74	347	28.0	0.89	0.58	1.35	.58
5-6	3	31.7	0.87	0.38	1.97		3	35.1	0.79	0.34	1.83	
PAI-1												
0-2	192	20.8	1.00				191	17.3	1.00			
3-4	277	22.2	0.89	0.65	1.23	.49	277	26.0	0.72	0.51	1.02	.06
5-6	8	23.6	0.80	0.42	1.50		8	39.2	0.52	0.26	1.03	

^1^Univariate analyses are unadjusted. Multivariate analyses are adjusted for age, sex, tumor grade, tumor stage, and lymph nodes.

^2^Median is the median years progression free as estimated from a parametric model.

^3^HR is the hazard ratio from a semiparametric (Cox proportional hazards) model. The 95% confidence limits (LCL and UCL) are shown.

^4^The expression p-values are for trend.

**Table 4b:** Progression Free Results for Muscle Invasive Bladder Cancer

	Univariate Analyses^1^						Multivariate Analyses^1^					
	n	Median^2^	HR^3^	LCL	UCL	p^4^	n	Median^2^	HR^3^	LCL	UCL	p^4^
Age (years)												
≤ 65	176	3.8	1.00				176	3.5	1.00			
> 65	248	2.4	1.30	1.04	1.63	.02	248	2.4	1.26	1.00	1.58	.05
Gender												
Female	130	2.0	1.00				130	1.9	1.00			
Male	297	3.4	0.71	0.56	0.89	.003	294	3.4	0.68	0.54	0.86	.001
Tumor Stage												
T2	382	3.0	1.00				381	2.9	1.00			
T3 or T4	44	2.6	1.06	0.75	1.49	.76	43	2.5	1.07	0.75	1.53	.72
Lymph Nodes												
N0 or N_X_	312	3.8	1.00				309	3.8	1.00			
N1	115	1.3	1.99	1.56	2.54	<.0001	115	1.3	2.01	1.57	2.57	<.0001
ANG												
0-2	318	3.1	1.00				315	3.0	1.00			
3-4	35	1.5	1.43	0.97	2.11	.07	35	1.5	1.40	0.95	2.07	.09
MMP-2												
0-2	190	4.0	1.00				189	3.9	1.00			
3-4	150	2.3	1.40	1.12	1.74	.003	148	2.2	1.43	1.13	1.80	.003
5-6	6	1.4	1.95	1.25	3.04		6	1.3	2.03	1.28	3.22	
p53												
0-2	140	2.4	1.00				140	2.5	1.00			
3-4	186	3.1	0.88	0.74	1.04	.13	183	2.9	0.91	0.77	1.08	.30
5-6	59	4.0	0.77	0.55	1.08		59	3.5	0.83	0.59	1.17	
Rb												
0-2	39	5.5	1.00				38	5.6	1.00			
3-4	311	2.8	1.40	1.02	1.93	.04	309	2.7	1.52	1.12	2.06	.007
5-6	11	1.4	1.96	1.04	3.72		11	1.3	2.31	1.26	4.23	
PAI-1												
0-2	143	3.9	1.00				142	3.4	1.00			
3-4	228	2.5	1.29	1.04	1.58	.02	226	2.6	1.19	0.96	1.48	.11
5-6	16	1.6	1.66	1.09	2.51		16	1.9	1.42	0.92	2.19	

^1^Univariate analyses are unadjusted. Multivariate analyses are adjusted for age, sex, tumor stage, and lymph nodes.

^2^Median is the median years progression free as estimated from a parametric model.

^3^HR is the hazard ratio from a semiparametric (Cox proportional hazards) model. The 95% confidence limits (LCL and UCL) are shown.

^4^The expression p-values are for trend.

**Table 5a:** Overall Survival Results for Non-Muscle Invasive Bladder Cancer

	Univariate Analyses^1^						Multivariate Analyses^1^					
	n	Median^2^	HR^3^	LCL	UCL	p^4^	n	Median^2^	HR^3^	LCL	UCL	p^4^
Age (years)												
≤ 65	183	16.7	1.00				181	15.4	1.00			
> 65	317	7.4	2.59	1.93	3.48	<.0001	317	7.5	2.41	1.78	3.26	<.0001
Gender												
Female	95	10.8	1.00				95	10.2	1.00			
Male	405	9.6	1.14	0.82	1.58	.44	403	9.7	1.07	0.77	1.49	.68
Tumor Grade												
Low	219	14.2	1.00				219	14.0	1.00			
High	279	7.2	2.10	1.62	2.73	<.0001	279	7.4	2.11	1.43	3.12	.0002
Tumor Stage												
Ta or Tis	253	12.4	1.00				252	9.3	1.00			
T1	247	7.7	1.67	1.30	2.15	.0001	246	10.3	0.88	0.61	1.27	.50
Lymph Nodes												
N0 or N_X_	487	10.2	1.00				486	10.0	1.00			
N1	13	2.7	4.03	2.19	7.41	<.0001	12	4.3	2.66	1.39	5.08	.003
ANG												
0-2	408	9.6	1.00				408	9.5	1.00			
3-4	29	12.6	0.75	0.42	1.35	.34	28	12.8	0.71	0.39	1.31	.28
MMP-2												
0-2	175	12.4	1.00				173	11.9	1.00			
3-4	281	9.1	1.40	1.06	1.84	.02	281	9.1	1.37	1.04	1.81	.03
5-6	2	6.6	1.95	1.12	3.37		2	7.0	1.87	1.08	3.26	
p53												
0-2	284	10.4	1.00				284	9.5	1.00			
3-4	173	9.7	1.08	0.87	1.35	.49	173	10.6	0.89	0.70	1.13	.34
5-6	24	9.1	1.17	0.75	1.82		24	11.7	0.80	0.49	1.28	
Rb												
0-2	114	17.9	1.00				114	14.7	1.00			
3-4	313	8.7	2.25	1.72	2.95	<.0001	312	9.2	1.76	1.34	2.31	.0001
5-6	20	4.2	5.07	2.95	8.72		20	5.8	3.08	1.79	5.32	
PAI-1												
0-2	192	13.3	1.00				191	12.0	1.00			
3-4	277	7.5	1.90	1.48	2.43	<.0001	277	8.1	1.61	1.23	2.09	.0004
5-6	8	4.3	3.60	2.20	5.89		8	5.5	2.58	1.52	4.38	

^1^Univariate analyses are unadjusted. Multivariate analyses are adjusted for age, sex, tumor grade, tumor stage, and lymph nodes.

^2^Median is the median years overall survival as estimated from a parametric model.

^3^HR is the hazard ratio from a semiparametric (Cox proportional hazards) model. The 95% confidence limits (LCL and UCL) are shown.

^4^The expression p-values are for trend.

**Table 5b:** Overall Survival Results for Muscle Invasive Bladder Cancer

	Univariate Analyses^1^						Multivariate Analyses^1^					
	n	Median^2^	HR^3^	LCL	UCL	p^4^	n	Median^2^	HR^3^	LCL	UCL	p^4^
Age (years)												
≤ 65	176	5.3	1.00				176	5.1	1.00			
> 65	248	3.3	1.41	1.12	1.77	.004	248	3.4	1.38	1.10	1.74	.006
Gender												
Female	130	2.8	1.00				130	2.7	1.00			
Male	297	4.7	0.70	0.55	0.88	.002	294	4.7	0.68	0.54	0.86	.002
Tumor Stage												
T2	382	4.1	1.00				381	4.1	1.00			
T3 or T4	44	3.7	1.07	0.75	1.52	.73	43	3.5	1.11	0.76	1.60	.59
Lymph Nodes												
N0 or N_X_	312	4.6	1.00				309	4.6	1.00			
N1	115	2.7	1.40	1.09	1.81	.008	115	2.7	1.40	1.09	1.80	.009
ANG												
0-2	318	4.1	1.00				315	4.0	1.00			
3-4	35	2.8	1.26	0.84	1.88	.26	35	3.1	1.20	0.80	1.80	.37
MMP-2												
0-2	190	5.5	1.00				189	5.4	1.00			
3-4	150	3.2	1.48	1.18	1.86	.0008	148	3.3	1.44	1.14	1.83	.003
5-6	6	1.9	2.19	1.38	3.46		6	2.0	2.08	1.29	3.33	
p53												
0-2	140	3.2	1.00				140	3.3	1.00			
3-4	186	4.2	0.84	0.71	1.00	.05	183	4.2	0.86	0.73	1.02	.09
5-6	59	5.6	0.70	0.50	0.99		59	5.2	0.74	0.53	1.05	
Rb												
0-2	39	6.5	1.00				38	6.6	1.00			
3-4	311	3.7	1.43	1.03	2.00	.03	309	3.7	1.48	1.07	2.03	.02
5-6	11	2.1	2.06	1.06	4.00		11	2.1	2.18	1.15	4.13	
PAI-1												
0-2	143	5.3	1.00				142	5.0	1.00			
3-4	228	3.5	1.37	1.11	1.70	.003	226	3.5	1.30	1.05	1.62	.02
5-6	16	2.3	1.88	1.23	2.88		16	2.5	1.69	1.09	2.62	

^1^Univariate analyses are unadjusted. Multivariate analyses are adjusted for age, sex, tumor stage, and lymph nodes.

^2^Median is the median years overall survival as estimated from a parametric model.

^3^HR is the hazard ratio from a semiparametric (Cox proportional hazards) model. The 95% confidence limits (LCL and UCL) are shown.

^4^The expression p-values are for trend.

## DISCUSSION

In this retrospective analysis, we demonstrated that the expression of MMP-2, RB and PAI-1 correlated with a reduction in recurrence-free survival and overall survival in patients with BCa. Our results also demonstrated the prognostic value of combining ANG, MMP-2, p53, RB and PAI-1 expression for all patients with BCa. Thus, patients whose bladder tumors express these biomarkers may benefit from early radical treatment and/or neoadjuvant or adjuvant therapies. To the best of our knowledge, this represents the largest and most comprehensive study to date demonstrating the prognostic significance of MMP-2, RB and PAI-1 in patients with BCa.

The biomarkers that comprised this prognostic signature (*i.e.,* ANG, MMP-2, p53, RB and PAI-1) have a varied range of ascribed functions including angiogenesis, breakdown of extracellular matrix, serine protein inhibitor, DNA binding and transcription factor. ANG and PAI-1 [22–26] have also been associated with angiogenesis. Angiogenesis, the development of new blood vessels from existing blood vessels, is essential for normal growth and the development of tissues and organs. Furthermore, in addition to the degradation extracellular matrix by MMP-2, recent studies have suggested that ANG and PAI-1 can breakdown the extracellular matrix [27, 28]. Degradation of the extracellular matrix allows cells to become more motile. Thus, these extracellular matrix-degrading proteins may work in conjunction with an increase in vasculature, thereby, increasing the probability that motile-invasive tumor cells may enter the circulation to disseminate to distant organs [29]. P53 has both transactivation and transrepression activity and, thus, controls the transcription of numerous genes [30–32]. RB also controls the expression of numerous genes although it does so primarily by recruiting transcription factors and chromatin remodelling proteins [33–35]. Furthermore, we have identified that ANG regulates MMP-2 [17], as well as PAI-1 and p53 expression, [36] while PAI-1 activity is controlled by p53 and RB [*unpublished data*].

Previous *in vitro* and *in vivo* analyses of ANG and PAI-1 began to unravel their potential contributions to BCa pathobiology. Briefly, there is clear evidence that ANG expression positively regulates MMP-2 expression *via* ERK1/2 signaling [17]. In addition, ANG regulates MMP-2 expression through two major pathways, a) p53 regulation *via* ERK pathway activation and b) induction of hypomethylation by DNMT3b down-regulation through the ERK pathway [36]. MMPs are known to affect extracellular matrix (ECM) remodeling, angiogenesis, apoptosis, epithelial-to-mesenchymal transition and cell proliferation and, thus, induce aggressive invasion and metastasis of different cancer types, including BCa [37]. Assessing the GEO database, we found that MIBC specimens had increased levels of ANG and MMP-2 compared to NMIBC. These findings suggest ANG may influence MMP-2 gene, which may be a mechanism to promote BCa growth and angiogenesis. Interestingly in the current study, ANG expression was not associated with tumor grade or tumor stage. Perhaps the inability to probe for ANG was due to the condition of the paraffin embedded tissue (*i.e.,* older sections may have some degradation of proteins). Thus, more research is needed to confirm ANG’s presence in human BCa. On the other hand, MMP-2 expression was greater in low-grade and low-stage BCa and was associated with a reduction in recurrence-free survival as well as reduction in overall survival.

PAI-1 is the primary inhibitor of tissue-type plasminogen activator (tPA) and urokinase-type plasminogen activator (uPA), and it acts to suppress tissue and plasma fibrinolysis *via* plasmin conversion [38]. We found that the down-regulation of PAI-1 led to a) an inhibition in cell proliferation and b) a potent arrest in the G_1_ to S phase of the cell cycle, a phenomena associated with reduction in cyclin D3/cdk4/6 and cyclin E/cdk2 and an increase in cell cycle inhibitors, p53, p21^Cip1/Waf1^, and p27^kip1^ [18]. Little attention has been given to PAI-1 in the human urinary bladder. For example, only two groups have reported PAI-1 levels for BCa patients. Urquidi *et al.* noted a significant increase in urothelial cell PAI-1 levels in patients bearing bladder tumors compared to non-tumor bearing patients [39]. Becker *et al.* reported significantly higher PAI-1 levels in tissue and plasma samples, but not in urine, from patients with BCa compared with controls [40]. Although the function of PAI-1 is complex, further investigation is warranted in the hope that it can provide insight into specific aspects of tumor biology and the identification of tumor cell vulnerabilities for therapeutic exploitation. Interestingly in the current study, higher PAI-1 expression was associated with high-grade compared to low-grade, while higher expression of p53 and RB were associated with high-grade and high stage disease. High PAI-1, p53 and RB were associated with a reduction in survival. Perhaps, the ability to demonstrate the difference in PAI-1 expression in this cohort compared to our previous cohort is related to the increase in the sample sizes of the current study. The high expression levels of p53 and RB have been well documented in BCa [41, 42]. In fact several studies have reported on the prognostic significance of p53 expression in bladder tumors. For example, Cordon-Cardo and others, noted an association between the p53-positive phenotype and disease progression (p < 0.001), as well as reduced survival (p < 0.001) in a small cohort comprised of 29 NMIBC [43]. Similarly in MIBC, Shariat and others, reported that alteration in p53 (or p21, pRB, p16) was independently associated with disease progression (p ≤ 0.038) and disease-specific survival (p *<* 0.039) in a small cohort of 127 MIBC treated with cystectomy [44]. Differences in outcomes associated with these studies and the current study may be related to our large, diverse cohort of 939 subjects. In the only phase III randomized study assessing the prognostic significance of p53 in BCa, Stadler and others, could not validate the prognostic significance of p53 in 521 patients undergoing adjuvant chemotherapy [45]. However, the current report begins to elucidate a specific pathway involving PAI-1, p53 and RB in human BCa. On the other hand, MMP-2 expression was greater in low-grade and low-stage BCa and was associated with a reduction in recurrence-free survival, as well as a reduction in overall survival.

Our findings in this report also have therapeutic implications regarding the potential utility of targeting these proteins directly. We and others have previously reported that blocking ANG, MMP-2, p53, RB or PAI-1 in bladder cell lines can significantly reduce cell proliferation/survival [17, 18], migration and invasion [18, 45, 46] and angiogenesis [17, 47–49]. Our current results, when combined with these previous observations, further strengthen the rationale of targeting MMP-2, RB and PAI-1 directly for treatment of BCa.

In summary, we have demonstrated in this study that MMP-2, RB and PAI-1 expression is correlated with poor prognosis of patients with BCa. Our findings support the hypothesis that targeting these three molecules may improve the survival of patients with BCa.

## MATERIALS AND METHODS

### Patients and clinicopathologic information

The study was performed after approval by the Western Institutional Review Board under a request of waiver of consent on archived pathologic specimens. Tissue microarrays associated with two independent patient cohorts were used in the current study. Cohort #1 was comprised of 587 patients diagnosed with BCa from 1979-2007 at Aarhus University Hospital (Aarhus, Denmark) [21], including 2 sub-cohorts; one with 223 primary Ta/T1 tumors from patients treated by transurethral resection of the bladder and one with 364 tumors from radical cystectomy patients. Cohort #2 was comprised of 352 patients diagnosed with BCa from 1984-2005 at Uppsala University Hospital (Uppsala, Sweden) [50]. Thus, samples from 939 patients were available for analysis. Demographic, clinical, disease and treatment characteristics are presented in Table 1. The median follow-up for the entire cohort was 5.37 years.

Follow-up data also included progression-free survival (PFS), cancer-specific survival (CSS) and overall survival (OS). The endpoints of recurrence-free survival and OS were calculated from the date of surgery to the date of the recurrence or last follow-up.

### Immunohistochemcal staining of tissue microarrays

Immunostaining was performed using standard protocols. TMAs were deparaffinized in xylene and rehydrated using graded percentages of ethanol followed by antigen retrieval with citric acid buffer (pH 6.0, 95°C for 20 min). The slides were treated with 3% hydrogen peroxide in water to block endogenous peroxidase activity. Staining for ANG, MMP-2, p53, RB and PAI-1 was conducted using mouse anti-human ANG antibody (C-1; 1:25 dilution in blocking buffer, Santa Cruz Biotechnology), rabbit anti-MMP-2 antibody (AB19167; 1:100 dilution in Biocare Background Sniper, EMD Millipore), mouse anti-p53 antibody (NCL-L-p53-DO7; 1:100 dilution in Biocare Background Sniper, Leica Biosystems), rabbit anti-Rb Antibody (C-15; 1:500 dilution in Biocare Background Sniper, Santa Cruz Biotechnology), and rabbit anti-PAI-1 antibody (HPA050039; 1:100 dilution in blocking buffer, Sigma-Aldrich), respectively. The antibodies' specificity information can be found in the manufacturers’ datasheets or our previous publication [20]. Biotin-labeled horse anti-mouse IgG (2 μg/ml in blocking buffer, Vector Laboratories) was used as the secondary antibody. Immunoreactive signals were amplified by the formation of avidin-biotin peroxidase complexes and visualized using 3, 3'- diaminobenzidine (DAB). Nuclear counterstaining was conducted with hematoxylin.

The expression levels of ANG, MMP-2, p53, RB and PAI-1 were scored by assigning a proportion score and an intensity score [17, 18, 51, 52]. The estimated proportion (0 = 0% of cells; 1 = 1% to 40%; 2 = 41% to 75% and 3 = 76% to 100%) and the average intensity (0 = none; 1 = weak; 2 = intermediate and 3 = strong) of immunoreactive tumor cells were assessed. The proportion and intensity scores were combined to obtain a total staining score for each protein, which ranged from 0 to 6. Thus, the protein expression levels were determined based on the total ANG, MMP-2, p53, RB and PAI-1 staining score as follows: none = 0, low = 1 or 2, moderate = 3 or 4, high = 5 or 6. Two investigators (OTMC, YS), who were blinded to the clinicopathologic data and clinical outcomes, scored each core. When there was a discrepancy in the scoring, a third investigator reviewed consensus scoring was obtained.

Human lung (SERPINE1, MMP-2), liver (ANG) and tonsil (p53, Rb) were used as positive controls, and omitting the primary antibody served as the negative controls.

### Statistical analysis

SAS 9.4 (Cary, NC) was used to perform the statistical analyses. The relationship between immunoexpression of the five targets and clinicopathological features were assessed with the Fisher’s exact test (crosstabs), log-rank tests (progression-free survival, cancer-specific survival and overall survival), and multivariate Cox proportional hazards regression (used to adjust for demographic and clinical parameters). All tests were 2-tailed.

## SUPPLEMENTARY MATERIALS FIGURE AND TABLES



## Abbreviations

BCa: bladder cancer
NMIBC: non muscle invasive bladder cancer
MIBC: muscle invasive bladder cancer
ANG: angiogenin
PAI-1: plasminogen activator inhibitor 1
TMA: tissue microarray
HR: hazard ratio
OS: overall survival
tPA: tissue plasminogen activator
uPA: urokinase plasminogen activator
CSS: cancer specific survival
PFS: progression free survival
DAB: 3, 3'- diaminobenzidine
