# Supplementary material for: Association of MMP-2, RB and PAI-1 with decreased recurrence-free survival and overall survival in bladder cancer patients
Source: Oncotarget. 2017 Sep 6;8(59):99707–21. doi: 10.18632/oncotarget.20686 (PMC5725126; doi:10.18632/oncotarget.20686)
Supplement: Supplementary file 1 [file oncotarget-08-99707-s001.pdf]

Association of MMP-2, RB and PAI-1 with decreased recurrence-free survival and overall survival in bladder cancer patients

SUPPLEMENTARY MATERIALS

1a

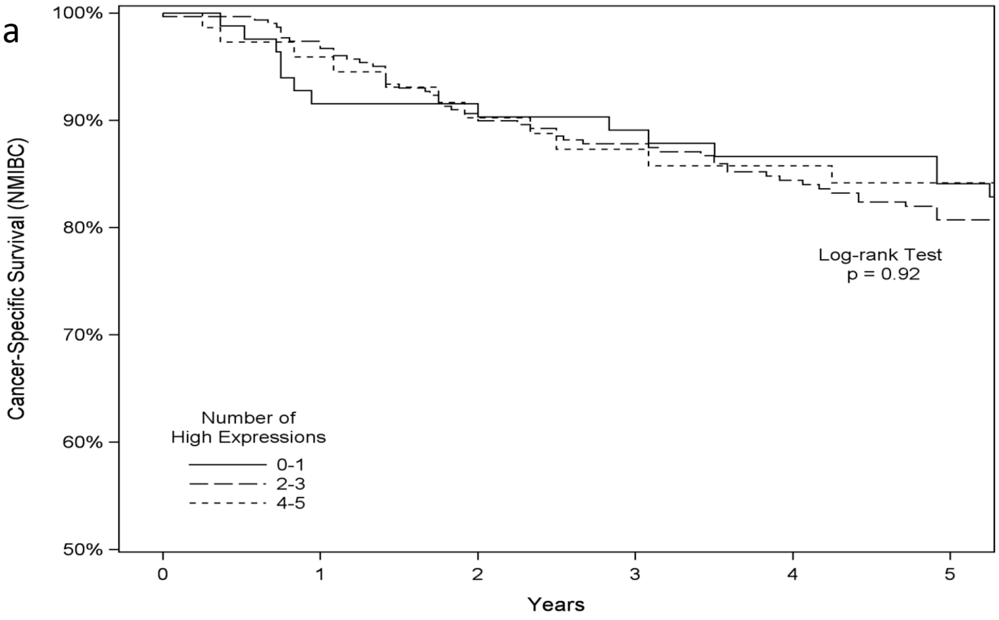

1b

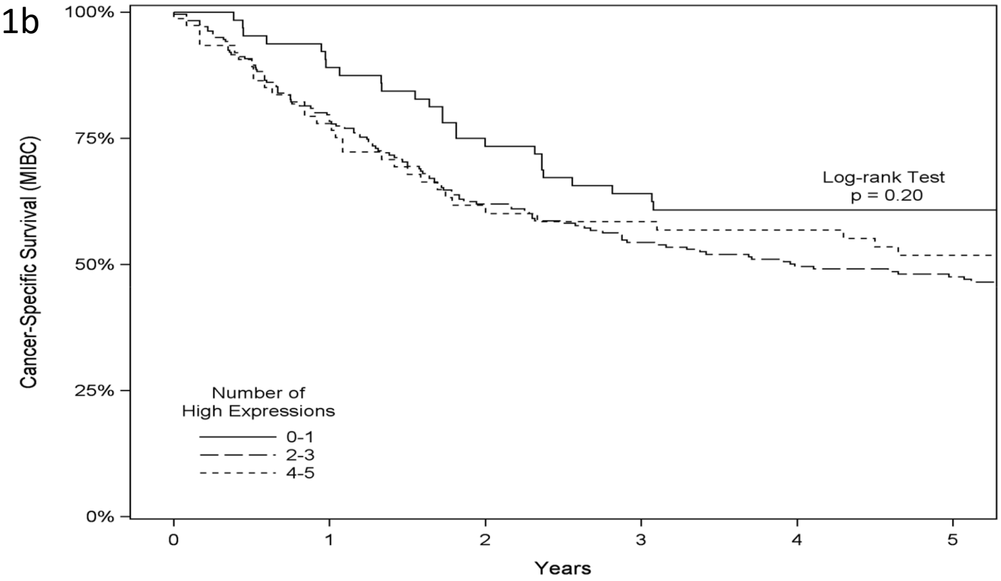

Supplementary Figure 1: Cancer-specific survival analysis of 939 patients with bladder cancer. Cancer-specific survival according to immunostaining status of ANG, MMP-2, p53, RB and PAI-1 in NMIBC **(A)** and MIBC **(B)**.

Supplementary Table 1a: Cancer-Specific Survival Results for Non-Muscle Invasive Bladder Cancer

	Univariate Analyses[1]						Multivariate Analyses[1]					
	n	Median[2]	HR[3]	LCL	UCL	p[4]	n	Median[2]	HR[3]	LCL	UCL	p[4]
Age (years)												
≤ 65	183	31.7	1.00				181	28.6	1.00			
> 65	317	19.0	1.52	1.03	2.26	.04	317	20.5	1.32	0.88	1.98	.19
Gender												
Female	95	28.9	1.00				95	24.5	1.00			
Male	405	22.7	1.25	0.77	2.05	.37	403	22.9	1.08	0.66	1.77	.77
Tumor Grade												
Low	219	43.5	1.00				219	35.9	1.00			
High	279	14.6	2.69	1.79	4.04	<.0001	279	16.4	2.02	1.09	3.73	.02
Tumor Stage												
Ta or Tis	250	38.9	1.00				250	25.7	1.00			
T1	250	14.8	2.39	1.63	3.51	<.0001	248	20.9	1.26	0.71	2.25	.43
Lymph Nodes												
N0 or NX	487	25.3	1.00				486	24.3	1.00			
N1	13	2.5	8.34	4.45	15.63	<.0001	12	3.9	5.97	3.04	11.70	<.0001
ANG												
0-2	408	25.2	1.00				408	24.5	1.00			
3-4	29	29.6	0.86	0.38	1.97	.73	28	36.0	0.69	0.28	1.71	.42
MMP-2												
0-2	175	29.7	1.00				173	28.8	1.00			
3-4	281	23.0	1.18	0.79	1.75	.43	281	22.1	1.20	0.80	1.80	.39
5-6	2	17.7	1.38	0.62	3.05		2	17.0	1.43	0.64	3.23	
p53												
0-2	284	27.6	1.00				284	22.7	1.00			
3-4	173	22.4	1.19	0.86	1.63	.29	173	24.6	0.93	0.65	1.31	.66
5-6	24	18.1	1.41	0.74	2.67		24	26.6	0.86	0.43	1.71	
Rb												
0-2	97	30.6	1.00				96	30.4	1.00			
3-4	347	25.8	1.22	0.75	1.99	.42	347	26.1	1.23	0.75	2.04	.41
5-6	3	21.7	1.50	0.56	3.97		3	22.3	1.52	0.56	4.17	
PAI-1												
0-2	192	24.5	1.00				191	22.3	1.00			
3-4	277	22.0	1.05	0.73	1.51	.77	277	23.9	0.91	0.62	1.33	.61
5-6	8	19.7	1.11	0.54	2.29		8	25.7	0.82	0.38	1.77	

[1]Univariate analyses are unadjusted. Multivariate analyses are adjusted for age, sex, tumor grade, tumor stage, and lymph nodes.
[2]Median is the median years cancer-specific survival as estimated from a parametric model.
[3]HR is the hazard ratio from a semiparametric (Cox proportional hazards) model. The 95% confidence limits (LCL and UCL) are shown.
[4]The expression p-values are for trend.

Supplementary Table 1b: Cancer-Specific Survival Results for Muscle Invasive Bladder Cancer

	Univariate Analyses[1]						Multivariate Analyses[1]					
	n	Median[2]	HR[3]	LCL	UCL	p[4]	n	Median[2]	HR[3]	LCL	UCL	p[4]
Age (years)												
≤ 65	176	9.1	1.00				176	8.9	1.00			
> 65	248	6.5	1.18	0.89	1.56	.26	248	6.9	1.15	0.87	1.53	.33
Gender												
Female	130	4.8	1.00				130	4.7	1.00			
Male	297	9.3	0.66	0.50	0.89	.006	294	9.5	0.65	0.48	0.87	.003
Tumor Stage												
T2	382	7.7	1.00				381	7.9	1.00			
T3 or T4	44	6.8	1.13	0.72	1.76	.60	43	6.0	1.25	0.80	1.97	.33
Lymph Nodes												
N0 or N_X	312	10.9	1.00				309	10.7	1.00			
N1	115	3.2	1.93	1.45	2.57	<.0001	115	3.1	1.92	1.44	2.55	<.0001
ANG												
0-2	318	8.5	1.00				315	8.4	1.00			
3-4	35	3.6	1.51	0.96	2.36	.07	35	4.2	1.48	0.94	2.32	.09
MMP-2												
0-2	190	10.2	1.00				189	10.0	1.00			
3-4	150	6.2	1.32	0.99	1.76	.06	148	6.3	1.32	0.98	1.77	.06
5-6	6	3.8	1.74	0.98	3.09		6	3.9	1.75	0.97	3.15	
p53												
0-2	140	6.1	1.00				140	6.6	1.00			
3-4	186	8.1	0.87	0.71	1.08	.22	183	8.0	0.92	0.74	1.14	.43
5-6	59	10.9	0.76	0.50	1.17		59	9.7	0.84	0.55	1.29	
Rb												
0-2	39	11.5	1.00				38	12.0	1.00			
3-4	311	6.8	1.32	0.88	1.97	.18	309	6.7	1.41	0.95	2.08	.09
5-6	11	4.0	1.74	0.78	3.88		11	3.7	1.98	0.91	4.32	
PAI-1												
0-2	143	10.4	1.00				142	9.4	1.00			
3-4	228	6.6	1.23	0.95	1.60	.12	226	6.8	1.16	0.89	1.52	.27
5-6	16	4.2	1.51	0.90	2.55		16	4.9	1.35	0.79	2.30	

[1]Univariate analyses are unadjusted. Multivariate analyses are adjusted for age, sex, tumor stage, and lymph nodes.
[2]Median is the median years cancer-specific survival as estimated from a parametric model.
[3]HR is the hazard ratio from a semiparametric (Cox proportional hazards) model. The 95% confidence limits (LCL and UCL) are shown.
[4]The expression p-values are for trend.
